# Monthly Summary

**Published:** 1896-11

**Authors:** 


### Monthly Summary.

INFILTRATION-ANESTHESIA IN DENTISTRY.—By C. H. Frink, D. D. S., Lake City, Florida.—The dental profession has been slow to approve of the use of local anesthetics for many reasons, chiefly among these the danger of systemic poisoning from cocaine, which nearly all contain in a greater or less degree.

It has been shown that the injection of simple water into the tissues, producing artificial edema, will induce local anesthesia. Dr. C. L. Schleich, an eminent surgeon of Germany, after a series of experiment found that a solution of three grains each of cocaine muriate and sodium chlorid, two-fifths of a grain of morphia muriate, and three fluid ounces of sterilized distilled water, had very marked anesthetic properties. The anesthetic is now being used with great success in all parts of the globe. It may be observed that this solution contains only one grain of cocain to the ounce of water, and, were it necessary, an ounce could be injected into the human system with impunity.

In using this anesthetic for the extraction of teeth, it is necessary to force as much of the fluid as possible into the adjacent tissues. In order to do this the needle should be inserted several times, keeping it, in most cases, parallel with the long axis of the tooth. This will allow deeper penetration of the needle and permit a greater quantity of the fluid being injected. A small crystal of cocaine, applied at the point of the first insertion of the needle, will render the injection of the solution painless. The effect of this is to produce a small circular area of a whitish appearance when thoroughly anesthetized. Each subsequent insertion should not be made outside of the anesthetized portion or wheel formed by the previous injection. When a sufficient quantity is inserted the tissue should assume a whitish appearance. The operation may be performed immediately.

The possible hypnotic effect of such a procedure before extraction is beneficial, as many patients feel much safer and are in a much better condition to undergo the operation when they observe that some step has been taken to lessen the pain.

This subject should be investigated by every conscientious dentist. We owe it to our patients that every method not endangering life be used for the alleviation of human suffering.—*Cosmos*.

---

GREEN-STAIN.—Use pyrozone to take off the stain, and milk of magnesia to prevent its return, the latter correcting the conditions that made the deposit of the pigment possible. —J. E. Line, in *Items of Interest*.

PREPARATION OF CAVITIES.—The preparation of cavities in teeth to receive gold does not admit of frail cavity-walls; does not admit of overhanging walls; does not admit of rough or irregular edges. I have particularly found that good, substantial walls with nicely polished edges are essential to success. I also advise, in approximal fillings, to cut back far enough to expose the point of junction between the gold and the tooth-substance, so as to admit of proper cleansing. In approximal fillings especially secure the point of contact between the two teeth in gold.

Regarding the insertion of gold into such cavities, I stand here as an advocate of the good old-fashioned method of hand-pressure; the motto ever before me being, that the great essential is not rocky solidity, but adaptation to the walls of the cavity with sufficient condensation to prevent disintegration. Mallets may be rapid, but time is nothing and the result everything, I verily believe that many fail from the effort with mallets to obtain unnecessary solidity. He who has cultivated strength in his fingers with that peculiar motion known to a hand-worker, and the drop of the wrist, can properly impact gold without mechanical adjuncts which only too frequently comminute the marginal edges of the cavity. Small pieces and small points, with not too much annealing, are the other requisites to success. Use, but be cautious in your use of matrics, and always contour your work.

The proper finishing of fillings I have found to require that the cavity should be filled to a little, a very little, over flush, and never so full as to be excessive, and then a thorough and persistent use of burnishers to the large exclusion, or very cautious use, of stones and files.

My last consideration is not the least in importance, but lies in the hands of the patient. The best work is liable to failure if the patient is careless in cleanliness. Having done all, every effort should be made to impress the need of this great adjunct upon the minds of those to whom the jewels are intrusted.—Dr. L. Ashley Faught, *Cosmos.*

FLEXIBLE RUBBER EDGES.—Vulcanite plates with flexible rubber edges for securing better atmospheric retention. Pack flasks in usual way with ordinary vulcanizable rubber, using draughtman's tracing cloth between the halves of the flask to permit separation. When the mold is accurately filled with the ordinary rubber, as much of this is trimmed away with scissors as it is desired to replace with the flexible plate rubber, Dougherty's or its equivalent, this packed and pressed to place and the case vulcanized for three hours or more. For upper plates the edge in the palatal region can be just back of the rugae, leaving the palate uncovered. The flexible edge on uppers needs to extend only from a point back of the buccinator muscle on one side across the palatal region to the same point on the opposite side. For lower dentures the flexible edge must constitute the entire periphery of the plate. Samples shown show atmospheric retention impossible to secure by plates made of hard rubber entirely. The model should be trimmed according to best judgment of operator, where flexible rubber is attached.—Dr. W. V. B. Ames, *Dental Review.*

---

THE SOLDERING IRON.—The soldering iron should be kept in perfect condition, otherwise good resul's cannot be obtained. A simple method of tinning the iron is, file copper down to clean surface and shape as desired. Place a small piece of tin or tinman's solder (half and half) on a slab of sal ammoniac, having iron thoroughly heated; rub it over slab, allowing it to come in contact with the tin till melted, then by a slight rotating motion iron will tin very readily. It is better to use tinman's solder for this purpose, as tin alone pits the iron. Care should be taken not to overheat iron in subsequent use, otherwise the tinned surface will be destroyed. When soldering, care should be taken to have clean surfaces. The soldering fluid (chloride of zinc), causing rapid oxidation, should not be applied till the iron is hot and ready for use, otherwise good union will not be obtained.—F. Messerchmitt, D. D. S., in *Items of Interest.*

LINING FOR RUBBER PLATES.—Aluminoid is a combination of rubber cement, any rubber dissolved in commercial chloroform, and aluminum powder. It is used to line the palatine surface of rubber plates.

To make: Dissolve rubber (such as we are furnished for vulcanizing) in commercial chloroform, it should be of about the consistency of thick molasses, incorporate with this about twice its bulk of albuminum powder; mix well. This gives you aluminoid. Invest wax upper plate in usual way, open and remove wax.

To Use. Coat your model with liquid collodion and apply the aluminoid, which should be thinned with chloroform so that it will flow nicely, like paint; glue four coats, allowing five minutes for each coat to dry; allow ten minutes or more for the last coat to dry. The case being packed, close and vulcanize; finish with brush and pumice, ending with chalk. The collodion and aluminoid are applied with camel's-hair brushes. The aluminum powder can be procured from dealers in art supplies. The cast is small, requires but little time to apply, and adds much to appearance and comfort of plate.—Dr. John G. Harper, *Dental Review.*

---

IN preparing a tooth for the reception of a porcelain crown (Logan or Richmond), before excising the natural crown, if you will take a piece of French rubber tubing, about one-eighth inch wide and a little smaller than the tooth to be crowned, carefully work it up on the neck of the tooth and as close to the gum as you can get without causing too much pain, allowing the patient to wear it for forty-eight hours, you can then face the root off under the gum line without laceration, hemorrhage or discomfort to your patient, which I consider quite an advantage in doing a nice piece of crown-work.

If natural crown is broken off build down with cement sufficient to give room to adjust rubber tube.—Dr. F. E. Judson, "Ohio Dental Journal."

DENTAL PAIN.—A very unusual case of pain following the extraction of a tooth has been brought to the attention of the Odontological Society of Great Britain, the patient, who was a female of some twenty-three years, having suffered pain, periostitic in character, from an upper third molar. The tooth was removed without difficulty, but the socket remained intensely painful for the next twelve days, notwithstanding the repeated applications of such remedies as strong carbolic acid, tincture of aconite, cocaine and hot poppy fomentations. During this period the socket granulated healthily except at its apex, where on examination a spot about the size of a pin's head was discovered, which appeared white in color, and caused, on being touched, the greatest agony. Thinking that the case was due to the exposure of the end of the nerve, the operator treated it by division of the nerve just below the surface of the wound, the result being completely satisfactory, as the pain ceased immediately when the operation was finished.

---

THE GREEK SURGEONS never take a stitch in a wound if they can help it, for there is a little insect which will do it for them. The black ant is a vicious fellow, and when his mandibles catch hold of anything he hangs on to it with the obstinacy of a bull-dog. When the surgeon has drawn the edges of the wound together he takes one of these black ants from a box, which he always carries about his person, with a pair of forceps, and the ant resents the instrusion by getting mad and grabbing the first thing he can find. The surgeon sees to it that it comes in contact with the edges of the wound, and the ant, with a grip that would surprise you, takes hold. Then the surgeon cuts off the fellow's head, for fear he will let go, which is never done after he is dead. When the aunt is defunct the mandibles do not relax in the slightest. All that is very odd and very interesting. But the time has not yet come when the black ant can be made useful in this country for that purpose.—"Items of Interest."

RESUSCITATION FROM ELECTRICAL SHOCK.—The use of electricity in the various arts and sciences is extending so rapidly that physicians are now liable to have calls to treat cases of unconsciousness or injuries accidentally caused by that agent. It is important to know that even very severe shocks often do not prove fatal. Persons may be apparently dead and yet may be resuscitated by the proper measures, timely employed.

A person apparently dead from lightning stroke or other electric shock is to be treated as one apparently drowned, excepting that there is no water in the respiratory, passages to complicate the treatment.

Artificial respiration should be employed, preferably by the method of rhythmical traction upon the tongue. Rhythmical compression of the region over the heart should be employed, about one hundred times per minute. Efforts to stimulate the functional activity of lungs and heart should be continued for an hour or more, as persons have been resuscitated when it seemed entirely hopeless.—"Medical World."

---

TIN AND GOLD FILLING.—I always keep tin and gold prepared in my cabinet and can fill an ordinary cavity with it in less time than it will take to prepare the amalgam, and I have a better filling when done. I would say this to those who wish to try it, that in making your filling, if you wish to change from tin and gold, to gold, do not anneal the gold until you have the surface of the combined materials well covered with unannealed gold. If you do you will work up a crumbly surface on the tin and gold, which will cause your filling to part at that point.—Dr. Robbins, *Dental Review*.

---

TO CLEAN AND REMOVE STAINS FROM TEETH.—Instead of using water with your pumice and iodin, or sulfuric acid. mix with 3 per cent. aqueous solution of pyrozone, and you will find it more effective and less deleterious in the teeth.—H. L. Harlan, in *Items of Interest*.

LOCAL ANESTHETIC.—Dr. J. R. Southworth, of Little Rock, Ark., uses the following as a local anesthetic instead of cocaine:

| | | |
|---|---|---|
| R. | Chloroform, | |
| | Tr. aconite - - | aa ʒ iij |
| | Tr. capsicum - - - | ʒ j |
| | Tr. pyrethrum - - - | ʒ ss |
| | Oil cloves - - - | ʒ ss |
| | Pul. camphor - - - | ʒ ss |
| Met. fl. sol. | | |

Directions.—Take a pledget of cotton large enough to envelop the tooth to be extracted, and the surrounding gum; saturate it with the fluid and apply to the tooth and gum, holding in contact—at same time holding with thumb and finger—for about half a minute; after lapse of half a minute, apply again in the same manner. Then extract the tooth.—*Items of Interest.*

---

THE SAVING of the pulp is important. In a child having a small decay in the first permanent molar, you should never use amalgam or gold for filling. You must get the confidence of children, and learn to handle them so as to accomplish the most good, and you can not do this with amalgam or gold. If you put in oxiphosphate till they are fourteen years of age, watching the tooth and renewing the filling as it needs it, the tooth will be perfectly preserved; if properly done he would defy any man to find decay. Then you can commence your good work with gold.—H. J. McKellops, in *Items of Interest.*

---

TREATMENT OF ABSCESS.—The injection of pure boiling water through the suppurative tract (with the same care in protecting the patient as in the use of escharotics), aided by the mechanical effect produced by the force of the syringe-injection, has proven more satisfactory than any of the chemical solutions I formerly used.—W. T. McLean, in *Items of Interest.*

SOMETIMES patients are unnecessarily subjected to long operations of great severity. I recall one instance where a lady went to a dentist, who is a brilliant operator, but who, unfortunately, has no consideration for the feelings of his patients. She had always submitted to dental operations without murmuring. She had perfect control of herself, and acquiesced in the judgment of her dentist. She was willing to stand any reasonable amount of severity, and had really at times suffered much at the hands of the dentist. But this time she was in the chair a little longer than usual, the nervous equilibrium overbalanced and the result was collapse. She left his office apparently in good condition, but when on the street began to succumb. She went to a drug-store, secured some brandy, and drank it as rapidly as she could and in considerable quantities, but it did not have any effect on her. She has never been the same woman since that operation and never expects to be.—Dr. Newkirk, "Dental Review."

---

THE HUMAN VOICE.—An inquiry having recently been instituted in London as to the greatest distance at which a man's voice could be heard without telephonic means, it appears that eighteen miles is the longest distance on record at which a man's voice has been heard—this, as related, having occurred in the Grand Canyon of Colorado, where one man shouted the name "Bob" at one end, and his voice was plainly heard at the other end, some eighteen miles away. Lieutenant Foster, on Parry's third Artic expedition, found that he could converse with a man across the harbor at Port Bowen, about one mile and a quarter distant; and Sir John Franklin said that he conversed with ease at a distance of more than a mile. Dr. Young records that at Gibralter the human voice has been heard at a distance of ten miles.

